# Prolonged Consumption of Sucrose in a Binge-Like Manner, Alters the Morphology of Medium Spiny Neurons in the Nucleus Accumbens Shell

**DOI:** 10.3389/fnbeh.2016.00054

**Published:** 2016-03-23

**Authors:** Paul M. Klenowski, Masroor R. Shariff, Arnauld Belmer, Matthew J. Fogarty, Erica W. H. Mu, Mark C. Bellingham, Selena E. Bartlett

**Affiliations:** ^1^Translational Research Institute and Institute for Health and Biomedical Innovation, Queensland University of TechnologyBrisbane, QLD, Australia; ^2^School of Biomedical Sciences, The University of QueenslandBrisbane, QLD, Australia

**Keywords:** binge-like consumption, long-term, medium spiny neuron, nucleus accumbens, sucrose

## Abstract

The modern diet has become highly sweetened, resulting in unprecedented levels of sugar consumption, particularly among adolescents. While chronic long-term sugar intake is known to contribute to the development of metabolic disorders including obesity and type II diabetes, little is known regarding the direct consequences of long-term, binge-like sugar consumption on the brain. Because sugar can cause the release of dopamine in the nucleus accumbens (NAc) similarly to drugs of abuse, we investigated changes in the morphology of neurons in this brain region following short- (4 weeks) and long-term (12 weeks) binge-like sucrose consumption using an intermittent two-bottle choice paradigm. We used Golgi-Cox staining to impregnate medium spiny neurons (MSNs) from the NAc core and shell of short- and long-term sucrose consuming rats and compared these to age-matched water controls. We show that prolonged binge-like sucrose consumption significantly decreased the total dendritic length of NAc shell MSNs compared to age-matched control rats. We also found that the restructuring of these neurons resulted primarily from reduced distal dendritic complexity. Conversely, we observed increased spine densities at the distal branch orders of NAc shell MSNs from long-term sucrose consuming rats. Combined, these results highlight the neuronal effects of prolonged binge-like intake of sucrose on NAc shell MSN morphology.

## Introduction

Over the last 40 years, there has been a documented rise in the consumption of sugar-sweetened beverages and foods containing added sugars (Nielsen et al., 2002; Popkin, 2010; Ng et al., 2012), with reports estimating that up to 75% of all foods and beverages contain high amounts of added sugars (Ford and Dietz, 2013; Bray and Popkin, 2014). During this period, there has also been a concurrent increase in the prevalence of obesity and type II diabetes, particularly among adolescents (Arslanian, 2002; Reinehr, 2013; Dabelea et al., 2014; Fryar et al., 2014). Recent studies have shown that overweight and obese children often consume high amounts of added sugar, however the contribution of high sugar containing diets to the increased incidence of overweight and obese children remains controversial (Hu, 2013; Bray and Popkin, 2014; Bucher Della Torre et al., 2015).

While a growing body of evidence indicates that the consumption of high sugar diets may, in part, contribute to weight gain among children and adolescents (Malik et al., 2010; Te Morenga et al., 2013; Bray and Popkin, 2014), less attention has been given to adverse non-metabolic consequences arising from excessive sugar intake. Interestingly, some common behavioral and psychological patterns often emerge amongst a subset of those who over eat and maintain high sugar containing diets. Most notable are the development of eating disorders including binge-eating, combined with the concurrent onset of psychological symptoms including lack of motivation and depression (reviewed in Sheehan and Herman, 2015). In addition, because binge-eating individuals often exhibit a loss of control and an inability to self-limit their sugar intake, it is likely that these behaviors arise as a result of neurological adaptations in brain regions that evaluate the hedonic value of highly palatable food (Saper et al., 2002; Lutter and Nestler, 2009; Kenny, 2011). This rationale is also supported by evidence in humans demonstrating that sugar and sweetness can cause cravings that are similar to those induced by addictive drugs such as alcohol and nicotine (Volkow et al., 2012).

Although the addictive properties of sugar are still speculative, these observations combined with studies demonstrating the contribution of excessive sugar intake to changes in reward circuitry and the development of addictive-like behaviors and emotional states in animal models (Avena et al., 2008; Benton, 2010; Ventura et al., 2014), warrants the need for further investigation. Previous studies in rodents have shown that intermittent access to sucrose alters the activity of several neurotransmitters within the mesolimbic system including dopamine, opioids and acetylcholine (Reviewed in Avena et al., 2008). Binge-like consumption of sucrose has been shown to facilitate dopamine release in the nucleus accumbens (NAc), similarly to drugs of abuse (Avena et al., 2008). Furthermore, we have shown that long-term consumption of sucrose using a 24 h intermittent access two-bottle choice paradigm (Simms et al., 2008) modulates nicotinic acetylcholine receptor (nAChR) expression in the NAc (Shariff et al., in press). Interestingly, we have also observed that nAChR compounds known to modulate dopamine and acetylcholine activity in the NAc, have different effects on sucrose consumption following short- and long-term intake (Shariff et al., in press).

While these studies have demonstrated similarities in the behavioral and neurochemical changes caused by intermittent access to sugar and drugs of abuse, it is not known whether these effects facilitate changes in neuronal morphology in the NAc. This is in contrast to substances of abuse including cocaine, amphetamine and nicotine which produce well characterized changes in the morphology of medium spiny neurons (MSNs) in the NAc, including increased spine density and altered dendritic complexity (Robinson and Kolb, 1999, 2004; Li et al., 2003; Crombag et al., 2005). Because we have previously shown that long-term exposure (12 week) to alcohol and sucrose using the intermittent two-bottle choice paradigm produces a differential response to pharmacotherapeutic interventions compared to short-term intake (4 weeks; Steensland et al., 2007; Shariff et al., in press), we assessed the effects of short- and long-term sucrose consumption on MSN morphology in the NAc. We allowed adolescent rats to consume sucrose in a binge-like manner for 4 (short-term) or 12 (long-term) weeks and then analyzed the morphology of NAc MSNs from short- and long-term sucrose consuming rats and compared this to age-matched controls who were given access to water only. Our results show that MSNs from the NAc shell are altered following long- but not short-term sucrose consumption, having reduced dendritic length, but increased distal dendritic spine density. Furthermore, we found the morphology of MSNs from the NAc core remained relatively intact following short- and long-term sucrose consumption. These results highlight a direct neurological consequence of long-term sucrose consumption in a binge-like manner. Furthermore, this data demonstrates the need for further studies aimed at elucidating the molecular and neurochemical changes that accompany the morphological restructuring of NAc shell MSNs induced by prolonged, binge-like sucrose intake.

## Materials and methods

### Ethics statement

All experimental procedures were carried out in accordance with the Australian Code for the Care and Use of Animals for Scientific Purposes, 8th Edition (National Health and Medical Research Council, 2013). The protocols were approved by the Queensland University of Technology Animal Ethics Committee and the University of Queensland Animal Ethics Committee.

### Animals and housing

Five-week-old (adolescent) male Wistar rats (Control: 176.4 ± 4.8 g; Sucrose: 178.3 ± 5.0 g) (ARC, WA, Australia), were individually housed in ventilated dual level Plexiglas® cages. The rats were acclimatized to the individual housing conditions, handling, and reverse-light cycle 5 days before the start of the experiments. All rats were housed in a climate-controlled 12-hr reversed light/dark cycle (lights off at 9 a.m.) room with standard rat chow and water available *ad libitum*.

### Intermittent-access two-bottle choice drinking paradigm

The intermittent access 5% sucrose two-bottle choice drinking paradigm (Simms et al., 2008) was adapted from Wise (1973). All fluids were presented in 300 ml graduated plastic bottles with stainless-steel drinking spouts inserted through two grommets in the front of the cage following the commencement of the dark light cycle. Weights of each bottle were recorded prior to bottle presentation. Two bottles were presented simultaneously: one bottle containing water; the second bottle containing 5% (w/v) sucrose. The placement of the 5% (w/v) sucrose bottle was alternated with each exposure to control for side preferences. Bottles were weighed 24 h after the fluids were presented, and measurements were taken to the nearest 0.1 g. The weight of each rat was also measured to calculate the grams of sucrose intake per kilogram of body weight. On day 1 of the drinking period, rats (*n* = 6–9) were given access to one bottle of 5% (w/v) sucrose and one bottle of water. After 24 h, the sucrose bottle was replaced with a second water bottle that was available for the next 24 h. This pattern was repeated on Wednesdays and Fridays. The rats had unlimited access to water on all other days. Binge-like consumption of sucrose resulted in an escalation in total sucrose intake (ml) over time (Supplementary Figure 1) and was accompanied by stable baseline drinking levels based on body weight [20 ± 5 g/kg of the 5% (w/v)] during the short-term [~4 weeks (13 drinking sessions)] and long-term [~12 weeks (37 drinking sessions)] drinking periods. A separate group of control rats (*n* = 6–9) were given access to water in both bottles (i.e., no sucrose) under the same conditions described above. The mean body weight of control and sucrose consuming rats at the end of short-term exposure was 405.7 ± 40.8 g and 426.4 ± 31.2 g respectively. At the end of long-term exposure, the mean body weight for control and sucrose groups was 578.8 ± 53.4 g and 600.2 ± 45.2 g.

### Golgi-Cox staining

Following the last drinking session rats were transferred from the animal facility to allow for processing of the brain samples at the histology facility at the School of Biomedical Sciences, University of Queensland (St Lucia, Australia). All approved measures were taken to reduce stress during transport, following which, rats were allowed to recover overnight. The next day, rats were sacrificed by sodium pentobarbital overdose (60–80 mg/kg, i.p. Vetcare, Brisbane, Australia) and intracardially perfused with ~300 ml artificial cerebro-spinal fluid that contained, (in mM): 130 NaCl, 3 KCl, 26 NaHCO_3_, 1.25 NaH_2_PO_4_, 5 MgCl_2_, 1 CaCl_2_, and 10 D-glucose. Each animal was then decapitated and the brain removed and incubated in the dark in Golgi-Cox solution that contained 5% potassium dichromate, 5% potassium chromate, and 5% mercuric chloride (all chemicals from Sigma-Aldrich) that was made fresh 3 days prior to sacrifice as described previously (Rutledge et al., 1969). Golgi-Cox stain incubation and post-processing methods were modified from Ranjan and Mallick (2010). Brains from short-term sucrose consuming animals were incubated for 6 days at 37°C, whilst brains from long-term sucrose consuming animals were incubated for 10 days, with one change to fresh Golgi-Cox solution after 4 days of incubation.

Following incubation, 300 μm coronal sections were cut using a vibrating Zeiss Hyrax V50 microtome (Carl Zeiss, Germany). Slices were then placed sequentially in 24-well plates filled with 30% (w/v) sucrose in 0.1 M phosphate buffered saline and processed as outlined in (Ranjan and Mallick, 2010). Briefly, sections were dehydrated in 50% ethanol for 5 min, then placed in 0.1 M NH_4_OH solution for 30 min, rinsed twice with distilled water for 5 min and placed in Fujihunt film fixer (Fujifilm, Singapore) for 30 min in the dark. The slices were then rinsed twice in distilled water for 2 min each and dehydrated in 70, 90, 95, and 100% ethanol twice for 5 min each. The sections were then cleared in CXA solution (1:1:1 chloroform:xylene:alcohol) for 10 min and mounted in DPX (Sigma-Aldrich) on Superfrost Plus slides (Menzel-Glaser, Lomb Scientific, Australia) and cover-slipped (Menzel-Glaser, Germany). The slides were left in the dark to dry at room temperature overnight.

### Neuronal selection and tracing within the nucleus accumbens

**Figure 1:**
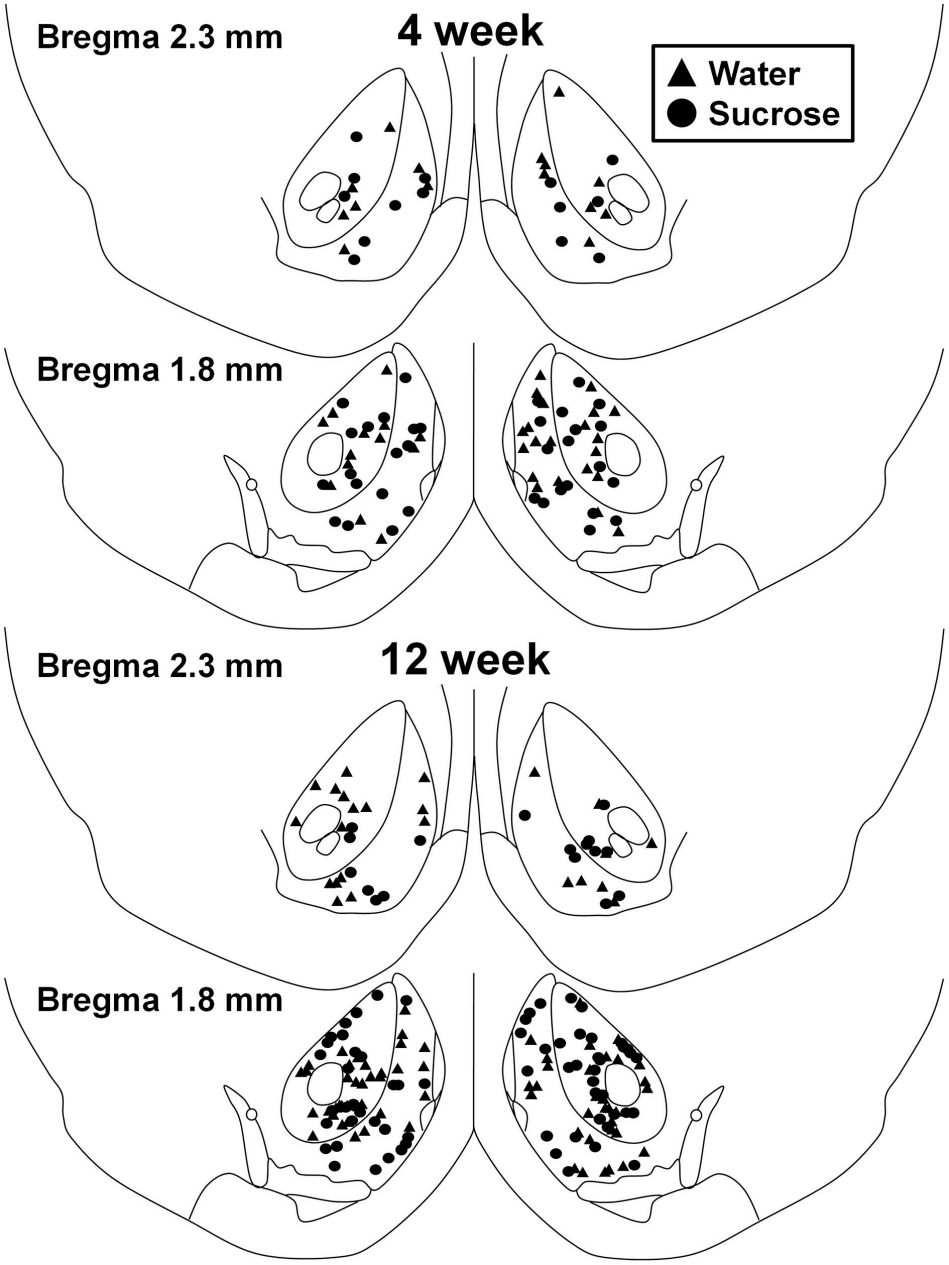
**Map showing locations of medium spiny neurons sampled from the nucleus accumbens core and shell of 4 and 12 week sucrose consuming rats and age-matched controls**. Top two panels show locations of neurons sampled from the nucleus accumbens core and shell of 4 week control (triangles) and sucrose (circles) animals. Bottom two panels show positions of neurons sampled from 12 week control (triangles) and sucrose (circles) animals.

**Figure 2:**
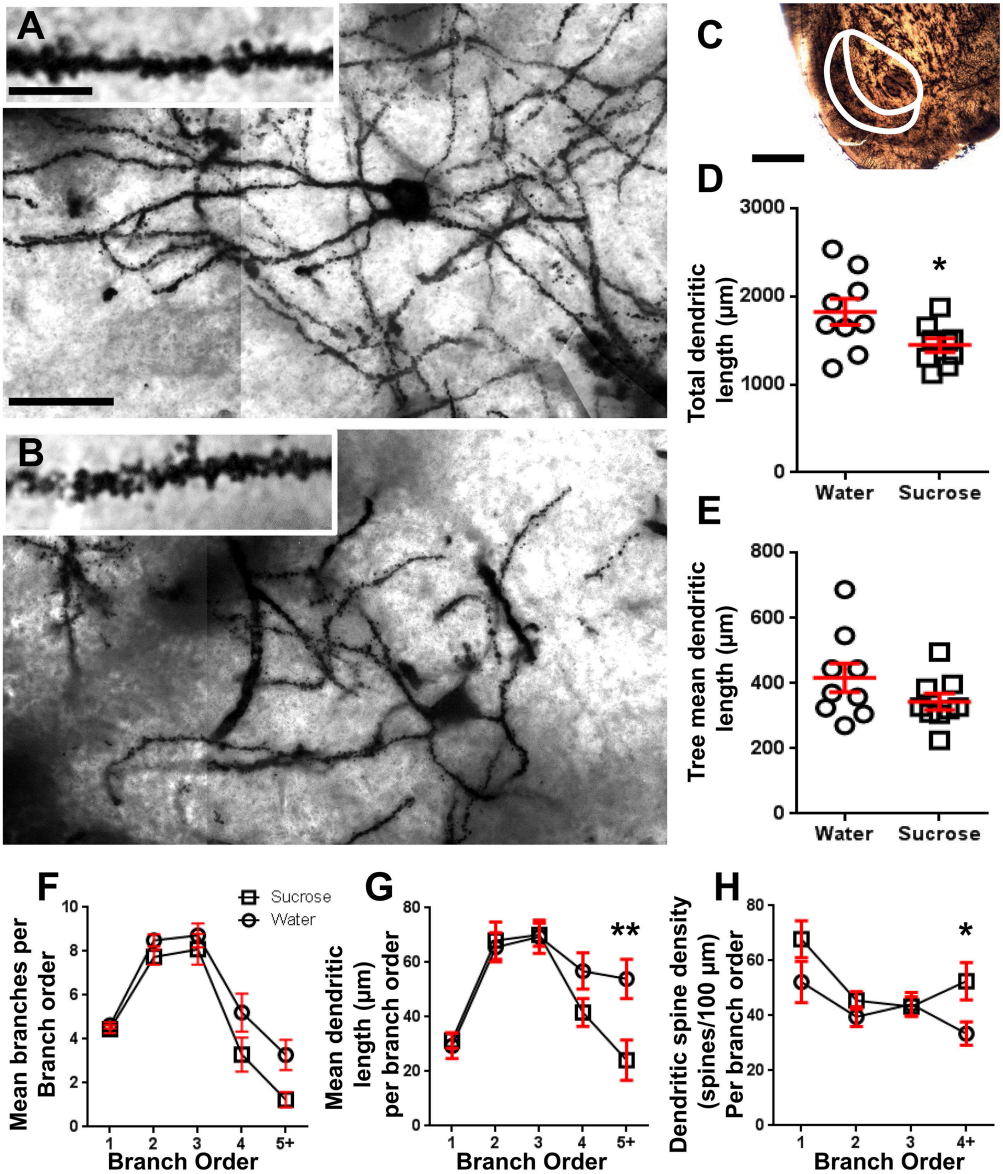
**Decreased dendritic arbor length and increased distal dendritic spine density of medium spiny neurons (MSNs) from the nucleus accumbens (NAc) shell of long-term sucrose treated rats compared to control rats. (A,B)** show representations of control (top) and long-term (12 week) sucrose (bottom) treated brightfield *z*-stack mosaics of Golgi-impregnated MSNs from the NAc shell (63x magnification). Inset of **(A,B)** shows control and long-term sucrose treated brightfield images of Golgi-impregnated MSN dendrites and dendritic spines from the NAc shell (100x magnification). **(C)** shows the anatomical regions that MSNs were sampled from in this study. **(D)** shows a scatter-plot of decreased total MSN dendritic arbor (mean ± SEM) from the NAc shell in long-term sucrose animals (squares) compared to controls (circles), unpaired students *t*-test, ^*^P < 0.05, *n* = 9; control and *n* = 9; 12 week sucrose. **(E)** shows a scatter-plot of unchanged mean MSN dendritic tree length (mean ± SEM) from the NAc shell in long-term sucrose animals (squares) compared to controls (circles), unpaired students *t*-test, *P* > 0.05, *n* = 9; control and *n* = 9; 12 week sucrose. Branch order analysis (mean ± SEM) of dendritic segment number per branch order **(F)**, mean dendritic length per branch order **(G)** and dendritic spine density per branch order **(H)**. Long-term sucrose consumption decreased dendritic length at distal branch orders (5+) and increased dendritic spine density at distal branch orders (4+) compared to controls **(G,H)**, two-way ANOVAs with Bonferroni post-tests, ^*^P < 0.05, ^**^P < 0.01, *n* = 9; control and *n* = 9; long-term sucrose. Scale Bars: **(A, B)** = 20 μm; inset of **(A, B)** = 10 μm; **(C)** = 1 mm.

Coronal slices between bregma +2.8 and +1.7 were surveyed for MSNs within the core and shell of the NAc, using the lateral ventricle and the anterior commissure as landmarks with the aid of a rat brain atlas (Paxinos and Watson, 2007) (Figure 1). The contour function in Neurolucida 7 (MBF Bioscience, VT, USA) was used to demarcate the NAc core and NAc shell in each slice (Figure 2). Between 2 and 9 neurons per region per animal were traced for dendritic length parameters using a 63x objective or for spine densities (reported as spines per 100 μm) using a 100x objective on a Zeiss Axioskop II (Carl Zeiss, Germany) using an automated *xyz* stage driven by Neurolucida® 7 software (MBF Biosciences, VT, USA). All tracing was performed in a blinded fashion with respect to treatment. Morphological parameters of Golgi-Cox impregnated neurons were analyzed in a manner similar to previous reports (Klenowski et al., 2015).

### Statistical analysis

Mean and standard error of the mean (SEM) were calculated for each data set with the animal as *n*, using the mean morphometry data from all the core or shell NAc MSNs (*n* = 7 for NAc shell and *n* = 6 for NAc core 4-week, *n* = 9 for 12-week groups). Where indicated, unpaired two tailed Student's *t*-tests or two-way ANOVAs with Bonferroni post-tests were conducted for all analyses involving the comparison of group means, using GraphPad Prism version 6.02 (GraphPad Software, San Diego, CA). Statistical significance was accepted at *P* < 0.05. All data in the results section are presented as means ± SEM. Percentage changes are calculated as relative to the control value.

## Results

### Medium spiny neurons from the nucleus accumbens shell have decreased dendritic length, decreased dendritic complexity but increased mean spine density at distal branch orders following long- but not short-term sucrose consumption

Following short-term (4 weeks) sucrose consumption, there were no significant differences in NAc shell MSN morphometric parameters (Table 1). There were also no significant differences between short-term sucrose consumption and water control NAc shell MSNs in analyses related to centrifugal branch order. Namely, dendritic segments per branch order (*P* = 0.4111), mean dendritic length per branch order (*P* = 0.5581) and mean spine density per branch order (*P* = 0.2977, two-way ANOVAs) were not significantly different between groups. A location map showing the approximate positions of the sampled neurons is shown in Figure 1.

**Table 1:** **General morphologic parameters of medium spiny neurons from the nucleus accumbens shell of short-term sucrose consuming rats and age-matched water controls**.

**Parameter**	**Water (*n*)**	**Sucrose (*n*)**	***P*-value**
Soma Volume (μm^3^)	2337±149 (7)	2216±132 (7)	0.5606
Total Dendrite Length (μm)	1640±159 (7)	1515±189 (7)	0.6217
Mean Tree Length (μm)	422±22 (7)	392±32 (7)	0.4510
Nodes	10.9±1.1 (7)	9.9±1.1 (7)	0.4223
Endings	14.3±1.4 (7)	14.7±0.7 (7)	0.8136
Spines Per 100 μm	54.5±5.0 (7)	57.1±2.5 (7)	0.6356

*All data presented as mean ± SEM. Two-tailed unpaired Student's t-tests*.

Following long-term (12 weeks) of sucrose consumption, the total dendritic arbor length of NAc shell MSNs were decreased by 21% compared to water consuming controls (Water: 1827 ± 148 μm, *n* = 9; Sucrose 1449 ± 78 μm, *n* = 9, ^*^P = 0.0384, two-tailed unpaired Student's *t*-test, Figure 2, Table 2). Comparison of the mean number of dendritic bifurcations (nodes) and dendritic endings between the water and sucrose groups revealed a reduced (although not significant) level of dendritic complexity in NAc shell MSNs (nodes: Water 12.9 ± 1.4 *n* = 9, Sucrose 10.1 ± 0.8 *n* = 9, *P* = 0.0879; endings: Water 17.9 ± 1.4 *n* = 9, Sucrose 14.8 ± 0.7 *n* = 9, *P* = 0.0657, two-tailed unpaired Student's *t*-test, Table 2). There was no change in soma volume (*P* = 0.9400), mean dendritic tree length (*P* = 0.1646) or total spine density (*P* = 0.3662) in NAc shell MSNs from long-term sucrose consuming rats compared to water controls. These morphometric parameters are detailed in Table 2.

**Table 2:** **General morphologic parameters of medium spiny neurons from the nucleus accumbens shell of long-term sucrose consuming rats and age-matched water controls**.

**Parameter**	**Water (*n*)**	**Sucrose (*n*)**	***P*-value**
Soma Volume (μm^3^)	4508±387 (9)	4453±599(9)	0.9400
Total Dendrite Length (μm)	1827±148 (9)	1449±78 (9)	^*^0.0384
Mean Tree Length (μm)	417±44 (9)	343±25 (9)	0.1646
Nodes	12.9±1.4 (9)	10.1±0.8 (9)	0.0879
Endings	17.9±1.4 (9)	14.8±0.7 (9)	0.0657
Spines Per 100 μm	40.4±3.3 (9)	45.0±3.7 (9)	0.3662

*All data presented as mean ± SEM*.

* *P < 0.05, two-tailed unpaired Student's t-tests*.

Following the characterization of the general dendritic morphology of long-term sucrose consuming NAc shell MSNs, we analyzed dendritic arborizations and spine densities with regard to their branch order characteristics. Our comprehensive assessment of the dendritic trees quantified the number of dendritic segments per branch order, the mean length of dendritic segments per branch order and mean spine density per branch order of NAc shell MSNs of water control and long-term sucrose consuming rats. A summary of the branch order data and analysis is presented in Table 3.

**Table 3:** **Branch order characteristics of medium spiny neurons from long-term sucrose and water drinking rats**.

**Branch order properties**	**Water (*9*)**	**Sucrose (*9*)**	**Adjusted *P*-value**
1st order branch segments	4.6±0.1	4.5±0.2	*P* > 0.9999
1st order mean branch segment length (μm)	25.9±3.5	31.4±2.8	*P* > 0.9999
1st order branch spine density	52.2±7.6	67.8±6.6	*P* = 0.2129
2nd order branch segments	8.5±0.3	7.7±0.3	*P* > 0.9999
2nd order mean branch segment length (μm)	65.2±6.0	68.0±6.8	*P* > 0.9999
2nd order branch spine density	41.3±3.6	45.5±3.2	*P* > 0.9999
3rd order branch segments	8.7±0.5	8.1±0.7	*P* > 0.9999
3rd order mean branch segment length (μm)	71.1±6.7	70.2±4.2	*P* > 0.9999
3rd order branch spine density	40.2±5.6	43.3±3.6	*P* > 0.9999
4th order branch segments	5.2±0.9	3.3±0.8	*P* = 0.0675
4th order mean branch segment length (μm)	56.9±6.6	41.6±5.1	*P* = 0.3308
4th order and above branch spine density	33.4±4.2	52.5±6.8	*P* = 0.0271^*^
5th order and above branch segments	3.3±0.7	1.2±0.3	*P* = 0.0566
5th order and above mean branch segment length (μm)	53.9±7.2	24.1±7.5	*P* = 0.0038^**^

All data presented as mean ± SEM. ^*^P < 0.05, ^**^P < 0.01, two-way ANOVAs with Bonferroni's post-test. P-values for condition are ^**^P = 0.0015 (segment number), ^*^P = 0.0318 (mean segment length) and ^*^P = 0.0124 (spine density).

The mean dendritic branch segment number per branch order of NAc shell MSNs was significantly reduced in long-term sucrose consuming rats compared to water controls (^**^P = 0.0015, two-way ANOVA). Bonferroni post-tests revealed a trend toward a reduced number of branch segments at 4th (Water: 5.2 ± 0.9, *n* = 9; Sucrose 3.3 ± 0.8, *n* = 9, *P* = 0.0675, Figure 2F, Table 3), and 5th order and above branch orders (Water: 3.3 ± 0.7, *n* = 9; Sucrose 1.2 ± 0.3, *n* = 9, *P* = 0.0566, Figure 2F, Table 3). The mean dendritic segment length per branch order of NAc shell MSNs was also significantly reduced in long-term sucrose consuming rats compared to water controls (^*^P = 0.0444, two-way ANOVA). Bonferroni post-tests showed a reduction of 55% at 5th order branches and beyond (Water: 53.9 ± 7.2 μm, *n* = 9; Sucrose 24.1 ± 7.5 μm, *n* = 9, ^**^P = 0.0038, Figure 2G, Table 3).

Branch order analysis showed a significant increase in the dendritic spine density of NAc shell MSNs of long-term sucrose consuming rats compared to controls (^*^P = 0.0124, two-way ANOVA). Bonferroni post-tests showed a spine density increase of 57% at distal 4th order branches and beyond (Water: 33.4 ± 4.2, *n* = 9; Sucrose 52.5 ± 6.8, *n* = 9, *P* = 0.0271^*^, inset of Figures 2A,B,H, Table 3). Representative images of overall MSN architecture and distal spine density (inset) are depicted in Figures 2A,B.

Taken together, these results indicate that short-term sucrose consumption has little effect on morphologic parameters of MSNs within the NAc shell. However, following prolonged consumption, there is a significant decrease in the neuronal arbor length and complexity, particularly in distal dendritic branches. Concomitant distal spine density increases are also apparent in NAc shell MSNs of long-term sucrose consuming rats.

### Medium spiny neurons from the nucleus accumbens core have reduced branching complexity after long- but not short-term sucrose consumption

Following short-term of sucrose consumption, there were no significant differences in NAc core MSN morphometric parameters (Table 4). There were also no significant differences between 4-week sucrose consumption and water control core MSNs in analyses related to centrifugal branch order. Namely, dendritic segments per branch order (*P* = 0.7717), mean dendritic length per branch order (*P* = 0.2096), and mean spine density per branch order (*P* = 0.3521, two-way ANOVAs) were not different between groups.

**Table 4:** **General morphologic parameters of medium spiny neurons from the nucleus accumbens core of short-term sucrose consuming rats and age matched water controls**.

**Parameter**	**Water (*n*)**	**Sucrose (*n*)**	***P*-value**
Soma Volume (μm^3^)	2774±293 (6)	2885±92 (6)	0.7491
Total Dendrite Length (μm)	1711±188 (6)	1793±132 (6)	0.7274
Mean Tree Length (μm)	416±25 (6)	444±37 (6)	0.5688
Nodes	11.1±1.0 (6)	11.9±0.8 (6)	0.5928
Endings	15.3±1.4 (6)	16.2±0.8 (6)	0.6014
Spines Per 100 μm	53.6±3.7 (6)	54.4±5.7 (6)	0.9142

*All data presented as mean ± SEM. Two-tailed unpaired Student's t-tests*.

Prolonged sucrose consumption also had no significant on NAc core MSN morphometric parameters (Table 5). The mean dendritic branch segment number per branch order of NAc core MSNs was significantly reduced in long-term sucrose consuming rats compared to water controls (^*^P = 0.0416, two-way ANOVA), however there were no significant differences in mean dendritic length per branch order (*P* = 0.0995) and mean spine density per branch order (*P* = 0.4888, two-way ANOVAs) between MSNs in the NAc core of long-term sucrose consuming rats compared to water controls. Taken together, our data shows the NAc core is not as responsive to long-term sucrose consumption compared to MSNs from the NAc shell region.

**Table 5:** **General morphologic parameters of medium spiny neurons from the nucleus accumbens core of long-term sucrose consuming rats and age matched water controls**.

**Parameter**	**Water (*n*)**	**Sucrose (*n*)**	***P*-value**
Soma Volume (μm^3^)	4569±230 (9)	4862±372 (9)	0.5031
Total Dendrite Length (μm)	1797±98 (9)	1775±120 (9)	0.8922
Mean Tree Length (μm)	404±32 (9)	398±40 (9)	0.8980
Nodes	13.2±1.2 (9)	11.4±0.7 (9)	0.2091
Endings	18.2±1.4 (9)	16.2±0.9 (9)	0.2456
Spines Per 100 μm	46.9±3.5 (9)	43.1±2.8 (9)	0.4130

*All data presented as mean ± SEM. Two-tailed unpaired Student's t-tests*.

## Discussion

The increased availability of highly sweetened food in the Western diet has not only contributed to the increased prevalence and economic burden of obesity and type II diabetes, it has also led to an onset of eating disorders such as binge eating (Swanson et al., 2011; Kessler et al., 2013; Davis, 2015). Although the addictive properties of sugars including fructose and sucrose remain speculative, there is a striking similarity in the behavioral and neural correlates that manifest as a result of over eating and prolonged drug use (Avena et al., 2008, 2011). In addition, sugar activates the brain's reward circuitry in a way that is similar to drugs of abuse (Volkow et al., 2012), and results from human studies suggest that sugar and sweetness can induce cravings that are comparable in magnitude to those induced by addictive drugs such as alcohol and nicotine (Volkow et al., 2012). We therefore used a model of binge-sucrose consumption in rats to determine the effects of short- (4 weeks) and long-term (12 weeks) sucrose consumption on neuronal morphology of MSNs in the NAc, a key component of the overlapping reward circuitry that is modulated by sugar and addictive drugs. We show that MSNs from the NAc shell of chronic long-term sucrose consuming rats have significantly decreased dendritic length and complexity, but increased distal dendritic spine density. Long-term sucrose consumption had no effect on the morphology of MSNs from the NAc core, while short-term sucrose consumption also had no significant effect on MSN morphology from the NAc core or shell. These results not only demonstrate a direct effect of prolonged binge-like sucrose intake on neuronal morphology of NAc shell MSNs, but they also highlight the potentially harmful consequences of the prolonged consumption of high sugar containing diets.

The NAc, which forms part of the ventral striatum, is comprised primarily of MSNs, which are morphologically characterized as medium-sized neurons with extensive dendritic arborisations and high spine density (Kemp and Powell, 1971; Graveland and DiFiglia, 1985; Rafols et al., 1989; Kawaguchi et al., 1990). Glutamatergic and dopaminergic neurons are the two primary afferent inputs to the NAc, primarily contacting the dendritic shafts and spines of MSNs (Groves, 1980; Kaiya and Namba, 1981; Groves et al., 1994). Specifically, the NAc shell and core receive glutamatergic input from functionally distinct cortical areas (Brog et al., 1993). The NAc shell is also innervated by excitatory afferents from subcortical regions such as the hippocampus, thalamus and basolateral amygdala (Brog et al., 1993; Wright and Groenewegen, 1995). Previous studies have demonstrated that these glutamatergic inputs play a pivotal role in motivation and goal-directed behaviors such as food and reward seeking (Maldonado-Irizarry et al., 1995; Kelley and Swanson, 1997; Reynolds and Berridge, 2003; Richard and Berridge, 2011). The other predominant input onto NAc MSNs is from dopaminergic afferents that project from the ventral tegmental area (Lindvall and Björklund, 1978; Veening et al., 1980; Kalivas and Miller, 1984). Interestingly, previous studies using similar models of intermittent sugar access have shown that the resulting binge-like consumption results in an increase in extracellular dopamine in the NAc similarly (albeit to a lesser extent) to drugs of abuse (Rada et al., 2005; Avena et al., 2006), and can modulate dopamine receptor expression (Colantuoni et al., 2001, 2002) in the NAc core and shell. Interestingly, binge-like consumption of sucrose causes an escalation in intake over time similarly to self-administration of drugs of abuse such as cocaine and heroin (Ahmed and Koob, 1998; Ahmed et al., 2000, 2003) which is associated with the development of an “addictive like” state.

Our analysis of branch order morphometry shows that the overall reduction in dendritic length of NAc shell MSNs caused by long-term sucrose intake, results primarily from reductions in the complexity of distal branch orders. We observed reduced distal branching (4th and 5th order and above branch orders) and significantly reduced mean length at 5th order and above dendrites, combined with increased spine densities at these branch orders. A common factor likely to influence this type of dendritic restructuring includes changes in synaptic connectivity and/or function (Russo et al., 2010). Previous studies have shown that glutamatergic synapses on MSNs are formed primarily on spines, particularly at distal dendrites (Groenewegen et al., 1999). Additionally, co-localization of dopamine and glutamatergic inputs from the prefrontal cortex (Sesack and Pickel, 1992), hippocampus (Totterdell and Smith, 1989; Sesack and Pickel, 1990), and amygdala (Johnson et al., 1994) have been observed on dendritic spines of MSNs. These observations combined with the increased spine density following long-term sucrose consumption seen in our study, support the formation of increased excitatory inputs. Therefore, the possibility arises where persistent effects caused by prolonged binge-like sucrose intake could facilitate increased excitatory synaptic activity at the distal dendrites of MSNs in the NAc shell. Consequently, reduction and/or retraction of distal dendrites may result via a synaptic homeostatic mechanism (Reissner and Kalivas, 2010), however this remains to be determined.

It is interesting to note that Crombag and colleagues showed that there was no spine density increase in the NAc shell following 4-week sucrose consumption via the nose-poke self-administration paradigm despite a more robust acquisition and a higher response rate to sucrose when compared with amphetamine (Crombag et al., 2005). Their observation of an absence of change in spine density at 4 weeks mirrors our findings. By contrast, however, our study demonstrates that following long-term (12 week) exposure to chronic sucrose consumption, there is a significant increase in distal spine density on the MSNs of the sucrose-experiences rats. Furthermore, our laboratory has previously shown that long-term (12 week) sucrose consumption facilitates a differential pharmacological response to pharmacotherapeutics that have been shown to modulate dopamine and acetylcholine responses at the level of the NAc (Shariff et al., in press). Taken together, this suggests that long-term (12 weeks and beyond) sucrose exposure, which is more accurately reflective of real-world scenarios, results in morphology adaptations at the level of the NAc.

In terms of drugs of abuse, repeated exposure to various drugs produces long-lasting changes in the structure of dendrites and dendritic spines. For example, amphetamines and cocaine both increase spine density in the NAc in both shell and core (Robinson and Kolb, 2004). Nicotine exposure has also been shown to increase spine density in the NAc shell. Conversely, morphine exposure leads to a decrease in spine density and dendritic branch complexity (Robinson and Kolb, 2004). In terms of long-term sucrose consumption, we observed an increase in spine density similar to amphetamine, cocaine and nicotine and opposite to the effect of morphine. However, unlike amphetamine and cocaine, but similar to nicotine, the increase of spine density on long-term exposure to sucrose is limited to the NAc shell. It is also interesting that changes in both dendritic branching (Robinson and Kolb, 1999) and spine density (Li et al., 2003) produced by amphetamine or cocaine are confined to distal dendrites of MSNs in the NAc, which reflects the findings in our study. Furthermore, and corroborative to the changes described above, sucrose consumption has also previously been shown to enhance excitatory synaptic strength onto accumbal dopamine neurons (Stuber et al., 2008b) as well as other components of the mesolimbic reward pathway (Stuber et al., 2008a; Chen et al., 2010). Taken together, this posits sucrose as a potent modulator of neuron morphology following prolonged heavy use, which is akin to the effects observed from drugs of abuse.

Although further investigations are required to uncover the cellular and synaptic mechanisms contributing to the morphological changes seen in this study, our results demonstrate significant neuronal effects engendered by long-term sucrose consumption. In particular, a consideration not examined in our study is whether the observed morphological effects of sucrose can also be elicited with non-caloric sweeteners such as saccharin. In this regard it is important to note that Lenoir and colleagues have shown that intense sweetness surpasses cocaine reward, be it generated by saccharin or sucrose (Lenoir et al., 2007). Furthermore, a recent study published by our lab (Shariff et al., in press) demonstrates that varenicline, a nicotinic acetylcholine receptor partial agonist reduced both sucrose and saccharin intake in rodents following the same long-term intermittent access regimen used in the present study. Interestingly, previous studies have shown similarities in the acute effects of non-caloric sweeteners such as saccharin and sucrose at the level of the NAc (Scheggi et al., 2013; Tukey et al., 2013; Carelli and West, 2014). However, further studies are need to determine whether non-caloric sweeteners can induce long-term effects similar to changes in the morphology of the NAc shell MSNs caused by long-term sucrose consumption reported here.

The lack of effect on NAc MSN morphology following short-term sucrose consumption, highlights the importance of implementing long-term studies to assess the impact of prolonged abuse of drugs or natural rewards like sucrose. In terms of dependence, not only are repeated cycles of binge intake and abstinence key components of the addiction cycle, a growing body of evidence has revealed that the transition to dependence is a progressive process that often occurs over an extended period of time. Although the addictive properties of sugars remain uncertain, the plausibility of addiction to other non-drug rewards such as sex, gambling and food is being increasingly investigated. The results from this study add merit to the hypothesis that sugars such as sucrose potentially have addictive properties following long-term, binge-like consumption. Our results also have implications for the growing number of children and adolescents who maintain unhealthy eating habits (high sugar consumption and binge eating) into adulthood. In line with the increased risk of developing metabolic effects it is also possible that neurological and psychiatric consequences affecting mood and motivation may also result from these behaviors.

## Author contributions

Participated in research design: PK, SB. Conducted experiments: PK, MS, AB, MF, EM. Data analysis: PK, MF, MS. Interpreted the data and contributed to writing of the manuscript: PK, MS, MF, EM, MB, SB. All authors read and approved the final manuscript for submission.

### Conflict of interest statement

The authors declare that the research was conducted in the absence of any commercial or financial relationships that could be construed as a potential conflict of interest. The reviewers SC, SA and handling Editor declared their shared affiliation, and the handling Editor states that the process nevertheless met the standards of a fair and objective review.

## Supplementary material

Supplementary Figure 1 **Sucrose intake and preference from 4 and 12 week sucrose consuming rats. (A,B)** show escalation in total sucrose intake (ml) over 4 and 12 weeks of exposure. **(C,D)** show high preference for sucrose over water during periods of sucrose presentation.
