# Supplementary figures and images for: Prolonged Consumption of Sucrose in a Binge-Like Manner, Alters the Morphology of Medium Spiny Neurons in the Nucleus Accumbens Shell
Source: Front Behav Neurosci. 2016 Mar 23;10:54. doi: 10.3389/fnbeh.2016.00054 (PMC4803740; doi:10.3389/fnbeh.2016.00054)

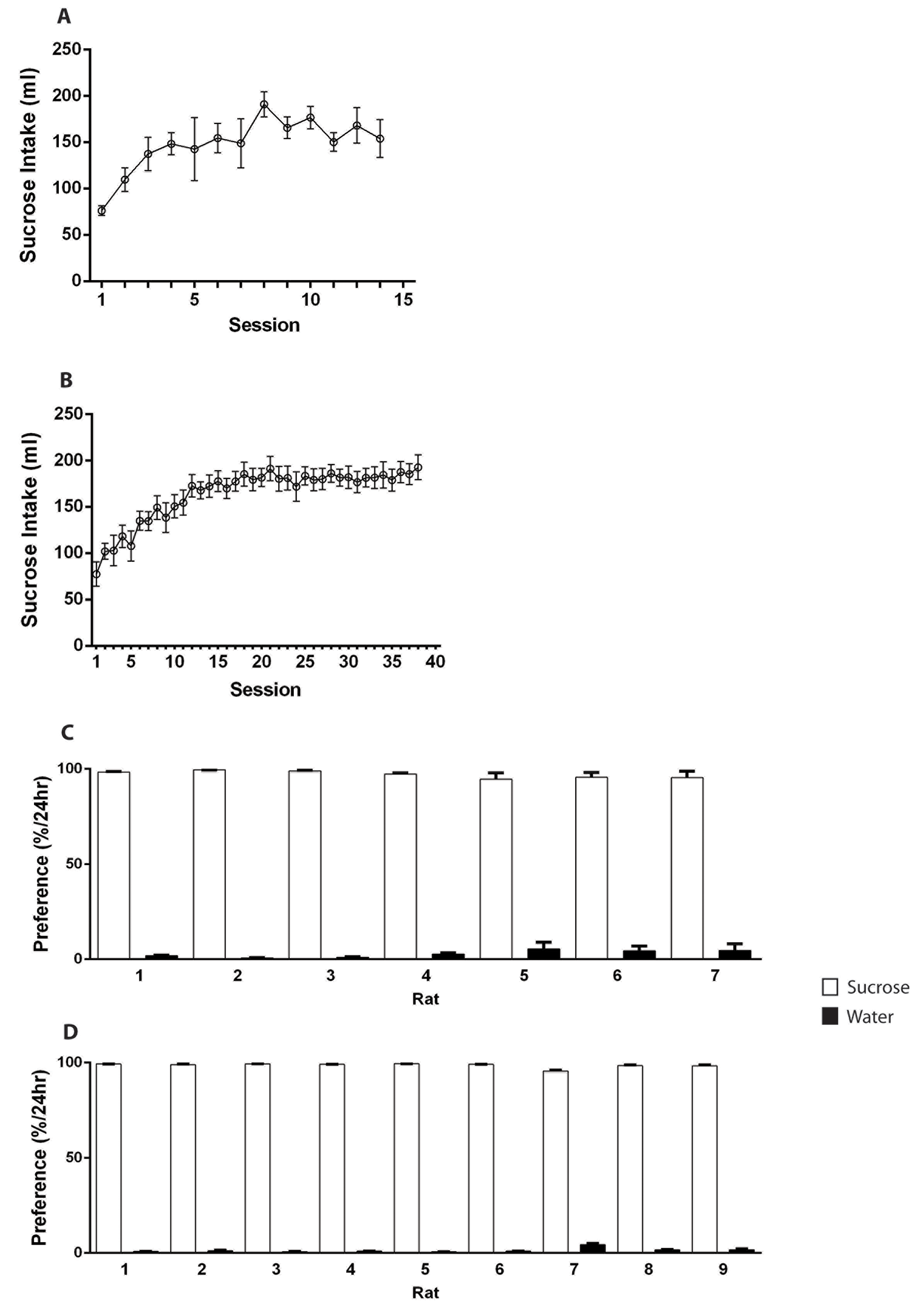

Supplement: Supplementary Figure 1 — Sucrose intake and preference from 4 and 12 week sucrose consuming rats. (A,B) show escalation in total sucrose intake (ml) over 4 and 12 weeks of exposure. (C,D) show high preference for sucrose over water during periods of sucrose presentation. [file Image1.TIF]
